# Occupational Health Hazards among Healthcare Workers in Kampala, Uganda

**DOI:** 10.1155/2015/913741

**Published:** 2015-01-31

**Authors:** Rawlance Ndejjo, Geofrey Musinguzi, Xiaozhong Yu, Esther Buregyeya, David Musoke, Jia-Sheng Wang, Abdullah Ali Halage, Christopher Whalen, William Bazeyo, Phillip Williams, John Ssempebwa

**Affiliations:** ^1^Department of Disease Control and Environmental Health, School of Public Health, College of Health Sciences, Makerere University, P.O. Box 7072, Kampala, Uganda; ^2^Department of Environmental Health Sciences, College of Public Health, University of Georgia, Athens, GA 30602, USA; ^3^Department of Epidemiology and Biostatistics, College of Public Health, University of Georgia, Athens, GA 30602, USA

## Abstract

*Objective.* To assess the occupational health hazards faced by healthcare workers and the mitigation measures. *Methods.* We conducted a cross-sectional study utilizing quantitative data collection methods among 200 respondents who worked in 8 major health facilities in Kampala. *Results.* Overall, 50.0% of respondents reported experiencing an occupational health hazard. Among these, 39.5% experienced biological hazards while 31.5% experienced nonbiological hazards. Predictors for experiencing hazards included not wearing the necessary personal protective equipment (PPE), working overtime, job related pressures, and working in multiple health facilities. Control measures to mitigate hazards were availing separate areas and containers to store medical waste and provision of safety tools and equipment. *Conclusion.* Healthcare workers in this setting experience several hazards in their workplaces. Associated factors include not wearing all necessary protective equipment, working overtime, experiencing work related pressures, and working in multiple facilities. Interventions should be instituted to mitigate the hazards. Specifically PPE supply gaps, job related pressures, and complacence in adhering to mitigation measures should be addressed.

## 1. Background

Worldwide, the healthcare workforce represents 12% of the working population [1]. Healthcare workers operate in an environment that is considered to be one of the most hazardous occupational settings [2–4]. In addition to the usual workplace related exposures, healthcare workers encounter diverse hazards due to their work related activities [5, 6]. In spite of this knowledge, the healthcare work environment continues to be neglected by governments and organizations [7]. A higher annual prevalence of back pain (77%) among healthcare workers compared to other occupational groups has been reported [8–11]. Back injuries are associated with a direct cost of $37,000 and an indirect cost ranging from $147,000 to $300,000 [8]. In fact, ergonomic related injuries pose a significant health risk to workers and yet it is the most prevalent occupational injury in healthcare industry [12]. Healthcare workers are exposed to blood-borne infections which usually expose them to diseases such as HIV, TB, and hepatitis B and hepatitis C [1]. Substantial morbidity and mortality among these workers inevitably lead to loss of skilled personnel and adversely impact healthcare services which are already strained in many low and middle income countries.

In sub-Saharan Africa, the scarcity of human resource for health is described as a humanitarian resource crisis due to significant emigration of trained professionals, difficult working conditions, poor salaries, low motivation, and high burden of infectious diseases, particularly HIV/AIDS [13–15]. Evidence from sub-Saharan Africa indicates that healthcare workers are frequently exposed to chemical, biological, physical, and psychosocial occupational hazards [6, 16]. They are constantly in contact with patients that expose them to infections and thus require proper protective measures to reduce their risk of acquisition of disease or injury. Data on occupational hazards among healthcare workers and their mitigation measures remain scarce in most of sub-Saharan Africa and Uganda in particular. Understanding the predisposing factors for occupational hazards among healthcare workers is needed to inform occupational health and safety policy and programs for healthcare workers. This study assessed the occupational hazards and their control measures in eight major hospitals in Kampala, Uganda.

## 2. Methods

### 2.1. Study Design and Setting

The study was cross-sectional in design and involved quantitative data collection methods. It was carried out in Kampala district, Uganda's capital city. There are over 873 health facilities in Kampala of which 26 are government owned, 22 private not for profit, and 825 private for profit [17].

### 2.2. Sampling

Eight (8) major hospitals were included in this study. These were purposively selected based on size and patient capacity. The selection ensured a combination of governmental, private for profit, and private not for profit facilities. These included Mulago Hospital and Butabika hospital, which are public facilities; Mengo hospital, Kibuli Muslim hospital, and Nsambya hospital, which are private not for profit; Kadic hospital, International Hospital Kampala, and Case Medical Centre which are private for profit facilities. To select the respondents, sampling proportionate to size was used to determine the number of healthcare workers to be interviewed from each hospital. At the hospital, all healthcare workers who were present at the facility were considered for the study. In cases where the number of workers present was higher than the required number of respondents, simple random sampling was used to select those to be interviewed.

### 2.3. Studied Population

The study population comprised a range of healthcare workers working in selected health facilities. These included doctors, nurses, clinical officers, and midwives.  Table 1 shows the general characteristics of the studied healthcare workers. Of the 200 participants, the majority were females (71.5%), were of age over 30 years (55.0%), were married (53.5%), were Christians (86.5%), and earned over 500,000 Uganda shillings (approx. US $200) per month (52.5%). Just over half were nurses (50.5%), 41% worked in private health facilities and 53.0% had practiced for five or more years. Most of them (70.5%) worked beyond their normal working time, 28.0% worked in more than one facility, 38% consumed alcohol, and 75.0% had less than 8 hours of sleep daily. Fifty nine (59) percent did not exercise frequently and most of them (83.5%) reported to have experienced job related pressures. None smoked tobacco.

### 2.4. Data Collection

A standardized structured questionnaire adapted from the National Institute of Occupational Safety and Health, US Center for Disease Control and Prevention, with modification to suit the local context consisting of both closed and open ended questions was used to collect the data.

The questionnaire collected data on the sociodemographic characteristics of the respondents including age, marital status, education level, and their work history. In addition, data was collected on the occupational health and safety practices of the healthcare workers, the hazards that they experienced in their work places, and the control measures in place to mitigate those hazards. The questionnaire was administered by a team of trained research assistants.

### 2.5. Data Management

Field inspection of questionnaire data was carried out daily after the field interview was conducted, and any errors were immediately verified and corrected. The quantitative data generated was entered in Epidata 3.02 and then entered into Stata 12.0 (StataCorp, College Station, TX) statistical software for analysis.

#### 2.5.1. Definition of Variables

- The occupational hazards were as follows. These hazards were broadly classified as biological and nonbiological. The broader classifications were generated as composite endpoints. Key questions asked included “Have you experienced any type of work-related illness/injury/trauma (major and minor)? If yes: What was the cause?” Twenty structured responses included (1) slips, trips, and falls; (2) physical, psychological, sexual, and/or verbal abuse; (3) cuts/wounds/lacerations; (4) burns; (5) fracture; (6) sharp-related injuries (i.e., needles, etc.); (7) radon from X-rays and so forth; (8) chemical spill; (9) noise; (10) direct contact with contaminated specimens/biohazardous materials; (11) bioterrorism; (12) musculoskeletal injuries such as muscle aches/strains/sprains, carpel tunnel syndrome; (13) blood-borne pathogens; (14) infectious diseases/infections; (16) airborne diseases; (17) vector borne diseases; (18) stress; (19) cross-contamination from soiled materials; and (20) nonionizing radiation. These responses were sorted to generated the composite classifications, biological or nonbiological. Consequently,
  - biological hazards were defined to include cuts/wounds/lacerations, sharp related injuries, direct contact with contaminated specimens/biohazardous materials, bioterrorism, blood-borne pathogens, infectious diseases/infections, airborne diseases, vector borne diseases, and cross contamination from soiled materials;
  - the nonbiological hazards were defined to include physical, psychosocial, and ergonomic hazards:
    - the physical hazards included slips, trips, falls, burns, fracture, radiation from X-rays, noise, and nonionizing radiation;
    - the psychosocial hazards included physical, psychosocial, sexual, and verbal abuse and stress;
    - the ergonomic hazards were musculoskeletal injuries such as muscle aches/strains/sprains and carpel tunnel syndrome.

### 2.6. Data Analysis

Data was analyzed using STATA 12 statistical software. To assess associations and independent predictors, we conducted binary and multivariate logistic regression modeling. The outcome variables used were experiencing either a biological or nonbiological hazard. Associations were run between the respondent characteristics and the outcome variables to obtain the significant associations. A *P* value of less than 0.05 was considered for a factor to be associated with experiencing the hazard.

### 2.7. Ethical Considerations

Ethical approval to carry out the study was obtained from Makerere University School of Public Health, Higher Degrees, Research and Ethics Committee, and the Uganda National Council of Science and Technology. Permission to conduct the study was obtained from the hospitals administration and each participant provided written informed consent.

## 3. Results

### 3.1. Biological and Nonbiological Hazards

Overall, half of the respondents reported experiencing an occupational health hazard. Among these, 39.5% experienced biological hazards while 31.5% reported experiencing nonbiological hazards.  Table 2 shows that the biological hazards mainly experienced by healthcare workers were sharp related injuries (21.5%), cuts and wounds (17.0%). The proportions of biological hazards (Table 3) were higher among healthcare workers who earned more than Ugx 500,000 per month (<500,000 (33.7%) versus >500,00 (44.8%)), worked in government hospitals (government (45.1%) versus others (32.3%)), never wore all necessary PPEs (wore all PPEs (30.4%) versus others (51.8%)), worked over time (yes (45.4%) versus no (25.4%)), worked in multiple health facilities (worked in multiple facilities (48.2%) versus single facilities (36.1%)), and experienced job related pressure (experienced job pressure (45.5%) versus others (9.1%)).

Among those that experienced nonbiological hazards (Table 2), the majority experienced stress (21.5%), physical, psychological, sexual, and/or verbal abuse (10.5%), and musculoskeletal injuries (10.5%). The proportions of nonbiological hazards (Table 3) were higher among females (male (26.3%) versus female (33.6%)), those older than 30 years (≤30 years (23.3%) versus >30 years (38.2%)), those who earned higher monthly incomes (≤500,000 (25.3%) versus >500,000 (37.1%)), those who worked in governmental facilities (government (39.0%) versus others (23.8%)), those with more than five-year duration in service (≤5 years (24.5%) versus >5 years (37.7%)), those who never wore all the necessary PPEs (wore all necessary PPEs (21.7%) versus others (44.7%)), worked over time (yes (36.9%) versus no (18.6%)), those who worked in multiple health facilities (multiple (41.1%) versus others (27.8%)), those who had less than 8 hours of daily sleep (<8 hours (34.0%) versus others (24.0%)), and those who experienced work related pressure (experienced pressure (36.5%) versus others (6.1%)).

### 3.2. Factors Associated with Exposure to Biological and Nonbiological Hazards

Table 4 shows the crude odds ratios for the associations between the respondents' characteristics and work related exposures. Biological hazards were associated with working in a government facility [COR = 2.21 (1.02–4.78), *P* = 0.043], not wearing all the necessary personal protective equipment [COR = 2.45 (1.37–4.39), *P* = 0.003], working overtime [COR = 2.44 (1.24–4.78), *P* = 0.009], and experiencing work related pressure [COR = 8.35 (2.45–28.4), *P* = 0.001]. Nonbiological hazards were associated with working in medical field for more than 5 years [COR = 1.87 (1.01–3.45), *P* = 0.045], working overtime [COR = 2.55 (1.22–5.34), *P* = 0.013], not wearing all necessary personal protective equipment [AOR = 2.45 (1.57–5.39), *P* = 0.006], and experiencing job related pressure [COR = 8.92 (2.06–38.57), *P* = 0.003].

At multivariate analysis (Table 5), the independent predictors for experiencing a biological hazard were not wearing necessary personal protective equipment [AOR = 2.34 (1.29–4.64), *P* = 0.006], working overtime [AOR = 2.65 (1.31–5.37), *P* = 0.007], and experiencing job related pressures [AOR = 8.54 (1.11–4.61), *P* = 0.001]. Meanwhile, the independent predictors for experiencing nonbiological hazards were not wearing all necessary personal protective equipment [AOR = 2.45 (1.29–4.64), *P* = 0.006], working overtime [AOR = 2.38 (1.10–5.14), *P* = 0.028], working in multiple health facilities [AOR = 2.26 (1.11–4.61), *P* = 0.024], and experiencing job related pressures [AOR = 9.69 (2.19–42.9), *P* = 0.003] (Table 5).

### 3.3. Control Measures for Occupational Health Hazards

We made an inquiry into the occupational measures in place to control occupational health hazards.  Table 6 shows the health facility and individual measures that were in place to control occupational health hazards. The major control measures provided by the health facilities were availing separate areas and containers to dispose medical waste (92.0%) and safety tools and equipment (90.0%). More than half (53.5%) of the health facilities provided health workers with personal protective equipment. Almost all healthcare workers had received HIV screening examination (97.0%) and 91.0% had received BCG vaccination. Regarding the hand washing practices, most health workers washed their hands before and after every procedure (79.5%) and after handling soiled materials (68.5%). Forty six percent of health workers washed hands when they were evidently dirty while slightly over half (53.5%) did so after using the toilet.

## 4. Discussion

This study highlights that half of respondents had experienced an occupational health hazard, mostly sharp related injuries and stress. The likely predictors for both biological and nonbiological hazards were not wearing all the necessary personal protective equipment, were working overtime, and were job related pressures. In addition, nonbiological hazards were predicted by working in multiple health facilities. The mitigation measures to control the hazards were mainly availing waste disposal facilities for the medical waste and provision of safety tools and equipment.

These findings are largely comparable to previous studies conducted in low and middle income countries. Ziraba in Uganda, Nsubuga in Uganda, Orij in Nigeria, De Castro in Philippines, and Adib-Hajbaghery in Iran reported that sharp related injuries and stress were the major health related hazards experienced by healthcare workers in their studies [6, 18–21].

In our study, we established that using all the necessary personal protective equipment was associated with reduced exposure to both biological and nonbiological hazards. This finding supports evidence by Hayden et al., who reported that use of PPEs reduced acquisition of illnesses in hospital settings [22]. Indeed, use and compliance with utilization of PPEs has for long been recognized as important infection control measure in the healthcare industry [23, 24] which should be emphasized to minimize exposure to occupational hazards.

In addition, we found that respondents who worked overtime had increased likelihood of experiencing both biological and nonbiological hazards. This is consistent with previous literature that reported increased risk of experiencing occupational hazards [21, 25–31]. Long working hours results in prolonged exposures to hazards and limited recovery time [32, 33] which translates into physiologic depletion that continues to the next workday [34]. Other studies have shown that working long hours is also associated with adverse health effects [31, 35, 36] and unhealthy behaviors [37]. This mode of work has also raised concern about patient safety [38]. We also found that respondents who experienced work related pressures were more likely to report occupational hazards. Work related pressures have been reported to have negative impacts including the compromise of patient care thus resulting to a diminished quality of life for both healthcare workers and patients [39, 40]. Another interesting finding although not significant at multivariate analysis was the association between working in a government health facility and experiencing occupational hazards compared to private health facilities. Reasons for this discrepancy may need to be explored further.

Although many health facilities provided waste disposal facilities for the medical waste and safety tools and equipment as control measures for occupational health hazards, simple measures like hand washing were not fully embraced. The proportion of health workers who reported washing hands after recommended procedures was lower than has been reported by previous studies [41, 42]. On the other hand, it was encouraging to establish that the majority of health workers had been screened for HIV and 8 in 10 health workers had been vaccinated against hepatitis B.

### 4.1. Study Limitations

This study was carried out in the major hospitals which limit generalizability to small and rural health facilities. The results could have been affected by recall bias as respondents were required to recall past experience. This being a cross sectional study, cause effect could not be established. Nevertheless, this study provides useful information on occupational health hazards in this low income context.

## 5. Conclusion

Healthcare workers continue to face several hazards in their workplaces. The factors associated with experiencing hazards include not wearing all necessary protective equipment, working overtime, experiencing work related pressures, and working in multiple facilities. Interventions should be instituted to mitigate the hazards. Specifically PPE supply gaps, job related pressures, and complacence in adhering to mitigation measures should be addressed.

## Figures and Tables

**Table 1:** Characteristics of studied health workers in major hospitals in Kampala, Uganda.

Characteristic	Category	Total *N* (%)
Overall		**200 (100.0)**

Sex	Male	57 (28.5)
	Female	143 (71.5)

Age	≤30 years	90 (45.0)
	>30 years	110 (55.0)

Marital status	Married	107 (53.5)
	Single	93 (46.5)

Religion	Christians	173 (86.5)
	Muslims	27 (13.5)

Head of household	No	97 (48.5)
	Yes	103 (51.5)

Cadre of health worker	Nurses	101 (50.5)
	Others	99 (49.5)

Monthly income	≤500,000	95 (47.5)
	>500,000	105 (52.5)

Type of health care facility	Faith based	48 (24.0)
	Government	70 (35.0)
	Private	82 (41.0)

Duration in service	<5 years	94 (47.0)
	≥5 years	106 (53.0)

Wearing all necessary PPE	Yes	115 (57.5)
	No	85 (42.5)

Working overtime	No	59 (29.5)
	Yes	141 (70.5)

Working in multiple facilities	No	144 (72.0)
	Yes	56 (28.0)

Alcohol consumption	No	162 (81.0)
	Yes	38 (19.0)

Has frequent exercise	No	118 (59.0)
	Yes	82 (41.0)

Daily hours of sleep	<8 hours	150 (75.0)
	≥8 hours	50 (25.0)

Pressure from job	No	33 (16.5)
	Yes	167 (83.5)

Tobacco smoking	No	200 (100)
	Yes	0 (0)

**Table 2:** Biological and nonbiological hazards experienced by health workers in major hospitals in Kampala, Uganda.

Hazards experienced by health workers	Frequency (*N* = 200)
	Yes (%)
Biological hazards	**79 (39.5)**
Sharp related injuries (such as needle sticks)	43 (21.5)
Cuts and wounds	34 (17)
Direct contact with contaminated specimens/biohazardous materials	21 (10.5)
Airborne diseases	18 (9.0)
Infectious diseases and/or infections	15 (7.5)
Others (blood borne pathogens, vector borne diseases, and bioterrorism)	15 (7.5)
Nonbiological hazards	**63 (31.5)**
Stress	43 (21.5)
Physical, psychological, sexual, and/or verbal abuse	21 (10.5)
Musculoskeletal injuries	21 (10.5)
Slips, trips, and/or falls	12 (6.0)
Fractures	10 (5.0)
Others (chemical spills, noise, burns, and radiations)	20 (10.0)

**Table 3:** Occupational health and safety hazards experienced by health workers in major hospitals in Kampala, Uganda.

Characteristic	Category	Biological hazard	Nonbiological hazard
		Yes (%)	Yes (%)
Overall	Total	79 (39.5)	63 (31.5)

Sex	Male	23 (40.3)	15 (26.3)
	Female	56 (39.2)	48 (33.6)

Age	≤30 years	35 (38.9)	21 (23.3)
	>30 years	44 (40.0)	42 (38.2)

Cadre of health worker	Nurses	40 (39.6)	37 (36.6)
	Others	39 (39.4)	26 (26.3)

Monthly income	≤500,000	32 (33.7)	24 (25.3)
	>500,000	47 (44.8)	39 (37.1)

Type of health care facility	Faith based	13 (27.1)	13 (27.1)
	Private	29 (41.4)	18 (25.7)
	Government	37 (45.1)	32 (39.0)

Duration in service	<5 years	36 (38.3)	23 (24.5)
	≥5 years	43 (40.6)	40 (37.7)

Wearing all necessary PPE	Yes	35 (30.4)	25 (21.7)
	No	44 (51.8)	38 (44.7)

Working overtime	No	15 (25.4)	11 (18.6)
	Yes	64 (45.4)	52 (36.9)

Working in multiple facilities	No	52 (36.1)	40 (27.8)
	Yes	27 (48.2)	23 (41.1)

Daily hours of sleep	<8 hours	64 (42.7)	51 (34.0)
	≥8 hours	15 (30.0)	12 (24.0)

Pressure from job	No	3 (9.1)	2 (6.1)
	Yes	76 (45.5)	61 (36.5)

**Table 4:** Crude odds ratios (COR) for the predictors of experiencing biological and nonbiological hazards among health workers in major hospitals in Kampala, Uganda.

Characteristic	Category	Biological hazards		Nonbiological hazards	
		COR [95% CI]	*P* value	COR [95% CI]	*P* value
Overall	Total	*N* = 79		*N* = 63	

Sex	Male	1		1	
	Female	0.95 [0.51–1.78]	0.877	1.41 [0.71–2.80]	0.32

Age	≤30 years	1		1	
	>30 years	1.04 [0.59–1.85]	0.873	2.03 [1.09–3.78]	**0.026** ^*^

Cadre of health worker	Nurses	1		1	
	Others	0.99 [0.56–1.75]	0.976	0.62 [0.34–1.13]	0.116

Monthly income	≤500,000	1		1	
	>500,000	1.59 [0.89–2.83]	0.11	1.75 [0.95–3.21]	0.072

Type of health care facility	Faith based	1		1	
	Private	1.90 [0.86–4.21]	0.112	0.93 [0.40–2.14]	0.868
	Government	2.21 [1.02–4.78]	**0.043** ^*^	1.72 [0.79–3.74]	0.169

Duration in service	<5 years	1		1	
	≥5 years	1.09 [0.62–1.94]	0.743	1.87 [1.01–3.45]	**0.045** ^*^

Wearing all necessary PPE	Yes	1		1	
	No	2.45 [1.37–4.39]	**0.003** ^**^	2.91 [1.57–5.39]	**0.001** ^**^

Working overtime	No	1		1	
	Yes	2.44 [1.24–4.78]	**0.009** ^**^	2.55 [1.22–5.34]	**0.013** ^*^

Working in multiple facilities	No	1		1	
	Yes	1.65 [0.88–3.08]	0.117	1.81 [0.95–3.45]	0.071

Daily hours of sleep	<8 hours	1		1	
	≥8 hours	0.57 [0.29–1.14]	0.115	0.61 [0.29–1.27]	0.19

Pressure from job	No	1		1	
	Yes	8.35 [2.45–28.4]	**0.001** ^**^	8.92 [2.06–38.57]	**0.003** ^**^

^*^ *P* < 0.05; ^**^ *P* < 0.01 indicates association between variables.

**Table 5:** Adjusted odds ratios (AOR) for the predictors of experiencing biological and nonbiological hazards among health workers in major hospitals in Kampala, Uganda.

Characteristic	Category	Biological hazards		Nonbiological hazards	
		AOR [95% CI]	*P* value	AOR [95% CI]	*P* value
Overall	Total	*N* = 79		*N* = 63	

Sex	Male	1		1	
	Female	1.09 [0.54–2.22]	0.796	1.32 [0.61–2.87]	0.488

Age	≤30 years	1		1	
	>30 years	1.23 [0.62–2.41]	0.553	2.02 [0.97–4.19]	0.059

Cadre of health worker	Nurses	1		1	
	Others	1.06 [0.56–2.01]	0.858	0.76 [0.38–1.51]	0.434

Monthly income	≤500,000	1		1	
	>500,000	1.52 [0.82–2.81]	0.180	1.74 [0.90–3.37]	0.097

Type of health care facility	Faith based	1		1	
	Private	1.88 [0.79–4.43]	0.151	0.93 [0.37–2.30]	0.872
	Government	1.96 [0.87–4.44]	0.105	1.43 [0.62–3.29]	0.398

Duration in service	<5 years	1		1	
	≥5 years	1.21 [0.49–2.98]	0.68	1.22 [0.58–2.82]	0.686

Wearing all necessary PPE	Yes	1		1	
	No	2.34 [1.27–4.28]	**0.006** ^**^	2.45 [1.29–4.64]	**0.006** ^**^

Working overtime	No	1		1	
	Yes	2.65 [1.31–5.37]	**0.007** ^**^	2.38 [1.10–5.14]	**0.028** ^*^

Working in multiple facilities	No	1		1	
	Yes	1.69 [0.88–3.29]	0.116	2.26 [1.11–4.61]	**0.024** ^*^

Daily hours of sleep	<8 hours	1		1	
	≥8 hours	0.57 [0.28–1.17]	0.125	0.56 [0.26–1.22]	0.145

Pressure from job	No	1		1	
	Yes	8.54 [2.48–29.4]	**0.001** ^**^	9.69 [2.19–42.9]	**0.003** ^**^

^*^ *P* < 0.05; ^**^ *P* < 0.01 indicates association between variables.

**Table 6:** Control measures to minimize exposure to occupational health and safety hazards among health workers in Kampala, Uganda.

Occupational health control measures	Frequency (*N* = 200)
	*N* (%)
Control measures provided by employers	
Safety education & training on all universal precautions	126 (63.0)
Safety tools, equipment, and machinery	180 (90.0)
Training on all machinery and equipment used	114 (57.0)
Training on how to wash hands	177 (88.5)
Personal set of personal protective equipment	107 (53.5)
Separate areas and containers to dispose medical waste	184 (92.0)
Individual protective measures	
BCG vaccination	183 (91.5)
Hepatitis A vaccination	64 (32.0)
Hepatitis B vaccination	156 (78.0)
Provision of postexposure prophylaxis	164 (82.0)
Ever received postexposure prophylaxis^*^	43 (21.5)
Received HIV screening examination	194 (97.0)
Hand washing practices	
After handling soiled materials	137 (68.5)
When hands are evidently dirty	92 (46.0)
Before and after meals	124 (62.0)
After using the toilet	107 (53.5)
After removing gloves	133 (66.5)
Before and after every procedure	159 (79.5)
Before and after handling each patient	136 (68.0)
After handling biological samples	136 (68.0)
Before and after handling hazardous materials	106 (53.0)

^*^Only received in case of exposure to HIV.
